# Endothelial cytochrome P450 -derived cholesterol limits angiogenesis

**DOI:** 10.1016/j.redox.2026.104289

**Published:** 2026-07-07

**Authors:** Pedro F. Malacarne, Melina Lopez, Souradeep Chatterjee, Niklas Herrle, Lisa Weiss, Stefan Günther, Timothy Osborne, Clemens Glaubitz, Tobias Schmid, Nina Kurrle, Frank Schnütgen, Dieter Lütjohann, Ralf P. Brandes, Flávia Rezende

**Affiliations:** a Goethe University, Institute for Cardiovascular Physiology, Frankfurt am Main, Germany; b German Centre of Cardiovascular Research (DZHK), Partner Site Rhein Main, Germany; c Max-Planck-Institute for Heart and Lung Research, Bad Nauheim, Germany; d Johns Hopkins University School of Medicine, Division of Endocrinology, Diabetes and Metabolism, Institute for Fundamental Biomedical Research, Johns Hopkins All Children's Hospital, Saint Petersburg, USA; e Goethe University Frankfurt, Institute for Biophysical Chemistry, Frankfurt am Main, Germany; f Goethe University Frankfurt, Institute of Biochemistry I, Frankfurt am Main, Germany; g Goethe University Frankfurt, University Medicine Frankfurt, Department of Medicine, Hematology/Oncology, Frankfurt am Main, Germany; h German Cancer Consortium (DKTK), Partner Site Frankfurt/Mainz, and German Cancer Research Center (DKFZ), Heidelberg, 69120, Germany; i Frankfurt Cancer Institute, Goethe-University Frankfurt, Frankfurt/Main, 60596, Germany; j University of Bonn, Institute for Clinical Chemistry and Clinical Pharmacology, Bonn, Germany

**Keywords:** Cholesterol, Cytochrome P450 oxidoreductase, SREBP2, Angiogenesis

## Abstract

The cytochrome P450 redox system is composed of a cytochrome P450 reductase (POR) and multiple CYP450 enzymes (CYP450). Of the CYP450 isoenzymes, CYP51A1 is essential for endogenous cholesterol biosynthesis. Elevated circulating cholesterol is a well-established risk factor for cardiovascular disease, however, the role of intracellular cholesterol synthesis in normal endothelial function remains unclear. To investigate this, we generated CRISPR/Cas9 knockouts of the cytochrome P450 reductase in primary human endothelial cells (EC) and studied an endothelial-specific, tamoxifen-inducible POR knockout mouse (ecPOR^−/−^). Deletion of POR led to the accumulation of lanosterol, the substrate of POR/CYP51A1, and a reduction in desmosterol. Functionally, POR deficiency promoted basal and VEGF-induced angiogenesis in spheroids and mouse aortic segments. Retinal angiogenesis was increased in ecPOR^−/−^ mice *in vivo*. Mechanistically, POR deletion activated the Sterol Regulatory Element Binding Transcription Factor (SREBP2) regulatory pathway, as shown by increased nuclear translocation of cleaved SREBP2 in EC and in *en face*-stained mouse aortae. Overexpression of nuclear SREBP2 in endothelial cells mimicked the angiogenic phenotype observed upon POR deletion. Conversely, double deletion of POR and SREBP2 normalized angiogenesis to levels of control cells. RNAseq of POR-deficient EC revealed an upregulation of PI3K-related signaling pathways and genes involved in cholesterol homeostasis, including enhanced expression of pro-angiogenic factors. In line with these findings, knockout of POR increased cellular PIP3 levels, AKT phosphorylation, and activation of downstream targets such as p70 S6 kinase. These findings demonstrate that inhibition of the endothelial POR/CYP51A1 axis impairs endogenous cholesterol synthesis, activates SREBP2, and enhances angiogenesis via PI3K/AKT/mTOR signaling, highlighting a critical and novel link between intracellular cholesterol metabolism and vascular growth.

## Highlights

• Endothelial deletion of POR/CYP51A1 blocks cholesterol synthesis promoting angiogenesis.
• Increased lipid uptake maintain total cholesterol levels in POR^−/−^ cells.
• POR^−/−^ activates SREBP2 signalling and nSREBP2 is sufficient to induce angiogenesis.
• Knockout of POR promotes the activation of pro-angiogenic kinases.

## 1 Introduction

Endothelial cells (EC) are important for angiogenesis, a process controlled by numerous growth factors and signaling pathways. Among them, the epoxyeicosatrienoic acids (EETs) stimulate endothelial cell proliferation and angiogenesis [[1], [2], [3], [4]]. EETs are endothelium-derived vasodilator autacoids produced from arachidonic acid by members of the cytochrome P450 enzyme family (CYP450). This enzyme system is also important for the oxidative metabolism of other endogenous substrates such as steroids and prostaglandins that potentially affect angiogenesis.

The CYP450 system comprises one reductase (cytochrome P450 reductase, POR), which is coded by a single gene, and more than 50 different CYP450 enzymes in humans and over 90 in mice [5]. POR is an FAD/FMN-containing NADPH-cytochrome P450 reductase that transfers electrons to CYP450 enzymes, which are heme-containing terminal monooxygenases. Thus the POR/CYP450 system is also known as a potential source of reactive oxygen species [6,7].

Given that the multiple CYP450 isoenzymes show overlapping specificity, our understanding of their endothelial *in vivo* function, particularly in angiogenesis, has been limited. The vascular role of EETs has been explored mostly in models of overexpression of CYP2C9 in EC [8], exogenous addition of EETs [9] or pharmacological inhibition of the EET-degrading soluble epoxide hydrolase (sEH) [10,11]. To overcome the limitations inherent to the complexity of CYP450 enzymes, we have recently developed an inducible, endothelial cell-specific knockout mouse of POR (ecPOR^−/−^) to generate a pan CYP450 system knockout mouse model. Endothelial deletion of POR reduced stores of EETs and induced vascular dysfunction and hypertension in mice [12].

Knockout of POR inactivates all microsomal CYP450 enzymes. CYP51A1, which participates in the synthesis of cholesterol by catalyzing the removal of the 14α-methyl group from lanosterol [13], is among the ones expressed in EC. Cholesterol is a fundamental lipid for cellular function, constituting up to 50 % of the outer plasma membrane composition. This high proportion shows its role in maintaining membrane structure and elasticity and in assisting key biological processes such as membrane trafficking and kinase signaling, which are important in the angiogenic process [13,14]. Extensive research has been performed on the negative role of cholesterol as part of lipoproteins in vascular diseases like atherosclerosis and statins, which inhibit 3-Hydroxy-3-Methylglutaryl-CoA Reductase (HMG-CoA-Reductase), are known to improve endothelial function [15]. The direct effects of statins on the endothelium are, however, mediated by pleiotropic effects unrelated to cholesterol synthesis but rather mediated by inhibition of protein geranylgeranylation [16]. Thus, the function of endogenous cholesterol production by the CYP450 system in EC remains poorly understood. Here we demonstrate that endothelial deletion of POR as well as of CYP51A1, which inhibits endogenous cholesterol synthesis, promoted angiogenesis.

## 2 Methods

### 2.1 Animals

Endothelial cell-specific, tamoxifen inducible mice knockout of POR (Por^flox/flox^-Cdh5-CreERT2^0/+^ termed as ecPOR^−/−^) were generated as previously described [12]. Control animals (CTR) were Por^flox/flox^-Cdh5-CreERT2^0/0^ littermates (i.e. no Cre expression). All animals had free access to chow and water in a specified pathogen-free facility with a 12 h day/12 h night cycle. All animal experiments follow the ARRIVE guidelines and all procedures conform to the guidelines from Directive 2010/63/EU of the European Parliament on the protection of animals used for scientific purpose. Experiments were performed in accordance to the German animal protection law under the protocol FU2006 and FU1188 and were carried out after approval by the local authorities (Regional council Darmstadt). Every mouse received an identification number for each experiment and the experimenter was blind for the genotype. Animal group sizes differ due to number of littermates. Control and knockout animals comprising both sexes were studied in paired fashion per experiment and the order was alternated daily. Mice were euthanized by terminal anesthesia with Isofluorane (10 % v/v) inhalation followed by organ harvesting. Mouse tissues were analysed *post mortem* and no surgeries have been performed that require analgesie.

### 2.2 Induction of the knockout of POR

One day old pups (ecPOR^−/−^ and their control counterparts) were injected intra-gastrically with 50 μL of tamoxifen at 1 mg/mL dissolved in sunflower oil for three consecutive days as previously described [12].

In adult mice (at least 8 weeks old), deletion of POR was induced by tamoxifen chow (CreActive TAM400 (400 mg/kg)) for 10 days. After tamoxifen feeding, there was a wash out time of two weeks before the experiments.

### 2.3 Neonatal angiogenesis

After inducing the knockout of POR by intra-gastric injection of tamoxifen, pups were euthanized by decapitation at post-natal day 7. Retinas were exposed and divided into four petals by incisions towards its center. The tissue was fixed with 100 % methanol at −20 °C overnight. Retinas were washed and treated with 0.1 % Triton X-100, 1 % bovine serum albumin (BSA) and 1 % donkey serum (Sigma, D9663). Next, retinas were stained with isolectin GS-IB4 (1:500, Thermo Fisher, I21411) overnight at 4 °C. Retinas were mounted on microscope slides (Thermo Scientific-Superfrost, 631-9483) using Dako Fluorescence Mounting medium (Agilent Technologies Inc., S3023). Images were acquired with a Zeiss LSM800 laser scanning microscope (Carl Zeiss Microscopy GmbH) under a 20× objective using the software ZEN (ZEN 3.1, Carl Zeiss Microscopy GmbH). Images were acquired using the tile mode. Quantification of the vascular network was performed using the software Angiotool64 (version 0.6a).

### 2.4 Aortic ring outgrowth assay

Aortic rings (1 mm) from ecPOR^−/−^ and their control counterparts were embedded in a gel mixture of rat tail collagen I (1.5 mg/mL, #354236, BD) 1x Medium 199 (#M0650, Sigma) and NaHCO_3_ (2.2 mg/mL) at 37 °C for 60 min. Endothelial basal medium (EBM) supplemented with 2.5 % autologous serum was added to the gels. Aortic rings were treated or not with murine VEGF (30 ng/mL, #450-32-10UG, PeproTech) and cultured for 7 days. Rings were fixed with 4 % paraformaldehyde (PFA), treated with 0.5 % Triton X-100 and 1 % BSA. Endothelial cells were stained with an anti-mouse CD31 antibody (1:200, #550274, BD). Images were acquired with a Zeiss LSM800 laser scanning microscope (Carl Zeiss Microscopy GmbH) using the tile- and Z-mode and sprouts were quantified using Angiotool64 (version 0.6a).

### 2.5 Cell culture

Pooled human umbilical vein endothelial cells (HUVEC) were purchased from PromoCell (#C12203, Heidelberg, Germany) and cultured in endothelial growth medium (EGM), consisting of EBM supplemented with 8 % foetal calf serum, 0.5 % penicillin/streptomycin (50 μg/mL), growth factors (EGF, bFGF, IGF, VEGF, #PB-C-MH-100-2199, PeloBiotech, Germany), heparin, l-glutamine but without hydrocortisone. For each experiment, at least three different batches of HUVEC at highest passage number 4 were used (different donors). For experiments with human aortic endothelial cells (HAEC), one vial of cells consisting of pooled donors was purchased and each “n” consisted of a different round of CRISPR. Lenti-X 293 T cells (Takara, 632180, Japan) were cultured in Dulbecco's Modified Eagle Medium High Glucose (Gibco) supplemented with 8 % FCS, penicillin/streptomycin (50 μg/mL of each) (#15140‐122, Gibco/Lifetechnologies, USA). All cells were cultured in a humidified atmosphere (5 % CO_2_, 37 °C).

### 2.6 CRISPR/Cas9 for cytochrome P450 reductase (POR), CYP51A1 and SREBP2

CRISPR/Cas9 knockout HUVEC and HAEC for POR, CYP51A1 and SREBP2 were generated with a lentiviral system as previously described [17]. Guide RNAs are listed in Table 1. Knockout efficiency was controlled by Western blotting.

**Table 1:** gRNAs for CRISPR/Cas9.

Gene	Sequence (sense)	Sequence (antisense)
NTC	CACCGTTCCGGGCTAACAAGTCCT	AAACAGGACTTGTTAGCCCGGAAC
POR	CACCGGTGTTCTACGGCTCCCAGA	AAACTCTGGGAGCCGTAGAACACC
CYP51A1	CACCGTTGGGGAGAAAATGTATGG	AAACCCATACATTTTCTCCCCAAC
SREBP2	CACCGGTGACCGGCTGTACCTGGG	AAACCCCAGGTACAGCCGGTCACC

Additionally, previously generated HUVEC reporter cell lines expressing cyan fluorescent protein (CFP) or yellow fluorescent protein (YFP) were also used for CRISPR/Cas9-mediated knockout of POR and NTC [18]. A mixed culture of NTC and POR^−/−^ reporter cell lines were used for a confrontational sprouting assay and also separated through fluorescence-activated cell sorting (FACS) for RNAseq.

### 2.7 Overexpression of nSREBP2

To constitutively overexpress the nuclear form of SREBP2, the human SREBP2 open reading frame was obtained from the plasmid pcDNA3.1-2xFLAG-SREBP-2 (plasmid #26807, Addgene, Cambridge, USA) and subcloned into the lentiviral vector pLVX (Clontech) for stable expression. This construct encodes the active nuclear form of SREBP2. Lentiviral particles were produced by transfecting HEK293T cells with the pLVX-nSREBP2 construct. Transduction of HUVEC was performed as described above for the lentiviral CRISPR/Cas9 procedure. Functional overexpression of nuclear SREBP2 was validated using a luciferase reporter assay as described below.

### 2.8 SREBP2 reporter gene assay

Reporter gene constructs were generated by cloning SREBP2 motif of LDL promoter and a point mutant negative control into pGL4.27 Luciferase Firefly. LDL receptor promoter forward sequence: “AAAATCACCCCACTGCAAACTCCTCCCCCTGC” and reverse complement sequence: “GCAGGGGGAGGAGTTTGCAGTGGGGTGATTTT”; point-mutant LDL receptor promoter forward sequence: “AAAAGAACCCCTATGCAAACTCCTCCCCCTGC” and reverse complement sequence: “GCAGGGGGAGGAGTTTGCATAGGGGTTCTTTT”. Overexpression of the respective plasmids was performed by electroporation with the Neon™ Transfection System (ThermoFisher) (1150 V; 30 ms; 1 pulse). The pGL4.35 luc2p/9x plasmid served as negative control. HAEC (100,000 cells) were electroporated and seeded onto 24 well plates in EBM for 4 h. Then, the medium was changed to EGM. 24 h after electroporation, luciferase activity was measured using the luciferase reporter gene assay (Promega, Cat. #E4530, USA) as described by the manufacturer. Luminescence was measured with Tecan Infinite M200 Pro (Switzerland).

### 2.9 nSREBP2 *en face*

The localization of SREBP2 in mouse aortae was detected with e*n face* staining using an antibody that was generated as previously described [19,20]. It exclusively detects the murine amino-terminal, nuclear-targeted domain of SREBP2 (nSREBP2). The thoracic aortae from CTR and ecPOR^−/−^ mice were dissected and 3 mm segments were cut open. They were fixed in 4 % PFA for 5 min followed by a permeabilization/blocking step with 1 % BSA, 0.1 % Triton X-100 and donkey serum for 4 h at room temperature. Next, the aortae segments were incubated with primary antibody at 1:50 at 4 °C overnight. The segments were then washed tree times with 0.1 % Tween in PBS followed by two washing steps with only PBS for 5 min each and then incubated with a secondary antibody anti-mouse (1:500) for 2 h. Vessel segments were mounted in Dako Fluorescence Mounting medium (Agilent Technologies Inc.) and images were acquired with a Zeiss LSM800 laser scanning microscope (Carl Zeiss Microscopy GmbH). Quantification was performed using ImageJ v.1.54.

### 2.10 Spheroid outgrowth assay

Spheroid outgrowth assays were performed as previously described [18]. Briefly, hanging drops were prepared by seeding 50,000 HUVEC per condition in EGM containing 20 % methyl cellulose onto hydrophobic dishes and culturing upside down overnight. The next day, spheroids were washed once with PBS and embedded into a collagen gel. Subgroups were treated with inhibitors or VEGF165 (30 ng/mL) for 16 h. Images were acquired with an Evos XL Core microscope (Life technologies). NTC and POR^−/−^ HUVEC reporter cells expressing CFP or YFP were also used for the confrontational spheroid outgrowth experiment. Images were acquired with the Evos XL Core or LSM 510 META (Zeiss). The quantitative analysis of sprout number and cumulative length was calculated with the ImageJ software version 1.48.

### 2.11 Cholesteromics from endothelial cells

HAEC were seeded onto 10 cm diameter plates and cultured in EGM. Once confluent, cells were washed with PBS and harvested with trypsin. Cell pellets were immediately frozen in liquid nitrogen. Cholesterol was measured by gas chromatography-flame ionisation detection (GC-FID) using 5α-cholestane as internal standard. The non-cholesterol sterols, cholesterol precursors and plant sterols were measured by GC-mass spectrometry-selected ion-monitoring (GC-MS-SIM) using epicoprostanol as internal standard together with the oxysterols by isotope dilution GC-MS-SIM using deuterium labeled oxysterols as previously described [21,22].

### 2.12 RT-qPCR

Total RNA isolation was performed with the RNA Mini Kit (Bio&Sell) according to the manufacturers protocol and reverse transcription was performed with the SuperScript III Reverse Transcriptase (#12574026, Thermo Fisher Scientific, Massachusetts, USA) using a combination of oligo(dT)23 and random hexamer primers (Sigma). The resulting cDNA was amplified in an AriaMX cycler (Agilent) with the ITaq Universal SYBR Green Supermix and ROX as reference dye (Bio-Rad, #1725125). Primers are listed in Table 2. Relative expression was calculated using the ΔΔCt method and genes were normalized to GAPDH.

**Table 2:** Primers for qPCRs.

Gene	Forward primer	Reverse Primer
POR	CGACGACGATGGGAACTTGG	GCTCGTACTGGCGAATGCTG
HMGCS1	AAGTCACACAAGATGCTACACCG	TCAGCGAAGACATCTGGTGCCA
HMGCR	GACGTGAACCTATGCTGGTCAG	GGTATCTGTTTCAGCCACTAAGG
LSS	CCGCAGGAAAACATTGCTGG	TGACTTGAAGGCCATGGAACG
GAPDH	TGCACCACCAACTGCTTAGC	GGCATGGACTGTGGTCATGAG
SREBP2	TGGCTTCTCTCCCTACTCCA	GAGAGGCACAGGAAGGTGAG
ANGPT2	TAAGGACCCCACTGTTGCTAAAGA	AATAATTGTCCACCCGCCTCCTC
HIF1α	GCTCATCAGTTGCCACTTCC	ACCAGCATCCAGAAGTTTCC

### 2.13 Immunoblotting

Cells were washed in cold PBS and scratched in the following lysis buffer was used: (pH 7.4, concentrations in mmol/L): Tris-HCl (50), NaCl (150), sodium pyrophosphate (10), sodiumfluoride (20), Triton X-100 (1 %), sodiumdesoxycholate (0.5 %), proteinase inhibitor mix, phenylmethylsulfonyl fluoride (1), orthovanadate (2), okadaic acid (0.00001). Homogenates were centrifuged (13.000 rpm, 10 min, 4 °C) and protein concentration was estimated with the Bradford method. Proteins (30 μg) were separated by SDS/PAGE, transferred by Western blotting and probed with the antibodies listed in Table 3. Western blotting analyses were performed with an infrared-based detection system (Odyssey, Licor, Bad Homburg, Germany).

**Table 3:** Antibodies.

Antibody	Catalog #	Company	Dilution
POR	SC-25270	Santa Cruz	1:500 WB; 1:200 IF
CYP51A1	13431-1-AP	Proteintech	1:500 WB
Phospho-AKT (Ser 473)	4058S	Cell signaling	1:1000 WB; 1:200 IF
SREBP2	10,007,663	Cayman	1:1000 WB
nSREBP2	Self-made	Osborne, T.F.	1:50 IF

### 2.14 FACS of CFP and YFP NTC and POR^−/−^ reporter cells

To separate the two cell populations, HUVEC expressing CFP or YFP were subjected to FACS using the Sony SH800S Cell Sorter (Sony Biotechnology). Mixed cultures were prepared containing NTC and POR^−/−^ cells expressing CFP or YFP, including reciprocal labelling conditions (NTC-CFP/POR^−/−^-YFP and NTC-YFP/POR^−/−^-CFP) mixed at a 1:1 ratio. Cells were detached, collected, and resuspended in FACS buffer to obtain a single-cell suspension prior to sorting.

Cells were first gated based on forward scatter (FSC-A) and side scatter (SSC-A) parameters to select the main cell population and exclude debris according to size and granularity. Doublets were excluded by gating FSC-A versus FSC-H to obtain single-cell events. Within the singlet population, CFP and YFP fluorescence signals were used to distinguish CFP-positive and YFP-positive cells. Fluorescence thresholds were defined based on the distribution of CFP and YFP intensities, and the respective populations were sorted into separate collection tubes using the Sony Cell Sorter Software (Version 2.1.6, Sony Biotechnology). Following sorting, cells were centrifuged at 1200 rpm for 4 min and resuspended in lysis buffer from the RNA Mini Kit (Bio&Cell). Flow cytometry data were analysed using FlowJo version 10.7.1.

### 2.15 RNA-sequencing

RNA isolation was performed as described in the RT-qPCR section. RNA, library preparation and sequencing was performed as reported previously [17].

Gene set enrichment analysis (GSEA) was performed using the Enrichr online platform (https://maayanlab.cloud/Enrichr/) using the GO Biological function database. Significance was determined by adjusted p-values.

Publicly available RNAseq data [23] (GEO accession: GSE202485) was downloaded and re-analysed to identify differentially expressed genes (DEGs) in POR-deficient brain microvascular endothelial cells (BMECs). Data was processed as for the HAEC RNAseq data, including normalization with DESeq2 and multiple testing correction using the Benjamini-Hochberg method (adjusted p-value <0.05). The commonly and significantly DEGs (padj <0.05) were compared to the HAEC-derived POR^−/−^ RNAseq data.

### 2.16 Phosphokinase array assay

Phosphorylation profiling was conducted using a commercially available phospho-kinase array kit (Proteome Profiler™ Array, Human Phospho-Kinase Array Kit, Cat.No. ARY003C, R&D) and followed the manufacturer's protocol. Briefly, 3 × 10^6^ cells were lysed with lysis buffer from the kit supplemented with aprotinin, leupeptin hemisulfate, and pepstatin (each at 10 μg/mL) A 300 μg of total protein lysate was incubated with the array membrane, which contained capture antibodies. After washing, membranes were incubated with a horseradish peroxidase (HRP)-conjugated detection antibody, followed by exposure to chemiluminescence reagents from the kit. Images were captured using a iBright FL-1500 ThermoFisher system. Densitometry was performed using ImageJ to quantify phosphorylation levels.

Extraction and detection of PIP2 (Phosphatidylinositol 4,5-bisphosphate) and PIP3 (Phosphatidylinositol 3,4,5-trisphosphate).

HAEC NTC and POR^−/−^ were grown in EGM and washed with ice-cold PBS, and lysed with 5 mL of 0.5 M trichloroaceteic acid. After scraping the cells, lysates were centrifuged at 3,000 rpm for 7 min at 4 °C. The pellet was washed twice with 3 mL of 5 % TCA/1 μM EDTA, followed by neutral lipid extraction with 3 mL methanol:chloroform (2:1) with vortexing for 10 min at room temperature. The supernatant was discarded, and the extraction was repeated.

Acidic lipids were extracted using 2.25 mL methanol:chloroform:12 M HCl (80:40:1) with continuous vortexing for 25 min. The supernatant was collected, mixed with 0.75 mL chloroform and 1.35 mL 0.1 M HCl, centrifuged, and the organic phase was collected (1.45 mL for PIP3, 0.05 mL for PI(4,5)P2).

Samples were divided into two separate aliquots and dried under nitrogen atmosphere. One aliquot was resuspended in 120 μL of PBS-Tween 0.3 % (for PIP3 measurements) and another in PBS (for PIP2 measurements). Samples were sonicated (10 min, ice-water bath), vortexed, and centrifuged and detected using the commercially available ELISA kits for PIP2 (BlueGene Biotech, Cat.No. E01P0009, Shanghai, China) and PIP3 (Biomatik Cat.# EKU11335, Ontario, Canada).

### 2.17 Proliferation assay

HUVEC (2,500 cells) were seeded onto a 96-well plate. Cells were grown in EGM containing IncuCyte Nuclight Rapid Red Dye in order to stain the Nuclei, according to the manufacturer's instructions. Proliferation was monitored by live cell imaging using the IncuCyte ZOOM software.

### 2.18 Migration scratch wound assay

HUVECs (NTC, POR^−/−^_ and CYP51A1^−/−^) were seeded in quadruplicates onto a 96 well ImageLock plate at a density of 50.000 cells/well. Cells were allowed to settle for 24h. The cellular monolayer was scraped in the center of the well in a straight line using a Scratch wound maker (SatoriusAG) and the media was immediately replaced with fresh EGM. The ImageLock plate was placed in the live-cell imaging Incucyte system. Images were acquired every 1h for a total period of 15h using the phase contrast imaging. Analysis was performed with the Incucyte software, thereby quantifying relative wound closure over time.

### 2.19 Filipin III staining in HAEC

Free cholesterol was assessed using filipin III staining. NTC and POR^−/−^ HAEC were cultured on 8-well chamber ibidi slides and fixed with 4 % PFA for 10 min at room temperature. After fixation, cells were washed with PBS and incubated with filipin III (50 μg/mL) for 30 min at room temperature in the dark. Following staining, cells were washed with PBS and imaged immediately using the LSM 800.

### 2.20 Fatty acid uptake assay

Fatty acid uptake was measured in NTC and POR^−/−^ HAEC as previously described [24]. Briefly, cells were plated onto Corning 96-well black-walled, clear-bottom plates at 40,000 cells/well. Cells received 2 μM BODIPY-C_12_ (ThermoFisher, D3822) (with 1 μM BSA) for 4 min, followed by 2 washing steps with 0.1 % BSA. Extracellular fluorescence was quenched with 0.08 % Trypan Blue. Intracellular fluorescence (bottom-read) was measured using a microplate reader at 488/520 nm (Tecan, Männedorf, Germany).

### 2.21 Assays for sterol and lipid uptake

HAEC (10.000 cells) were seeded onto 96-well plates. To assess cholesterol uptake, cells were incubated with NBD-cholesterol (5 μM, Thermofisher, N1148) in EGM and loaded onto the IncuCyte live-cell imaging system (Satorius, Göttingen). NBD-cholesterol accumulation was monitored over 9 h using the IncuCyte by acquiring images in the green fluorescence channel. The green fluorescent area per well was quantified using the IncuCyte ZOOM software.

### 2.22 Statistics

Data are represented as mean ± standard error of the mean. Calculations were performed with Prism 9.2.0. The latter was also used to test for normal distribution and similarity of variance. In the case of multiple testing, unless otherwise stated Bonferroni correction was applied. For multiple group comparisons, analysis of variance followed by post hoc testing was performed. Individual statistics of dependent samples were performed by paired *t*-test, of unpaired samples by unpaired *t*-test, and, if not normally distributed, by the Mann-Whitney *U* test as indicated. P values of <0.05 were considered significant. Unless otherwise indicated, n indicates the number of individual experiments or number of mice.

## 3 Results

### 3.1 Deletion of POR in endothelial cells increases angiogenesis in mice and in cells

**Fig. 1:**
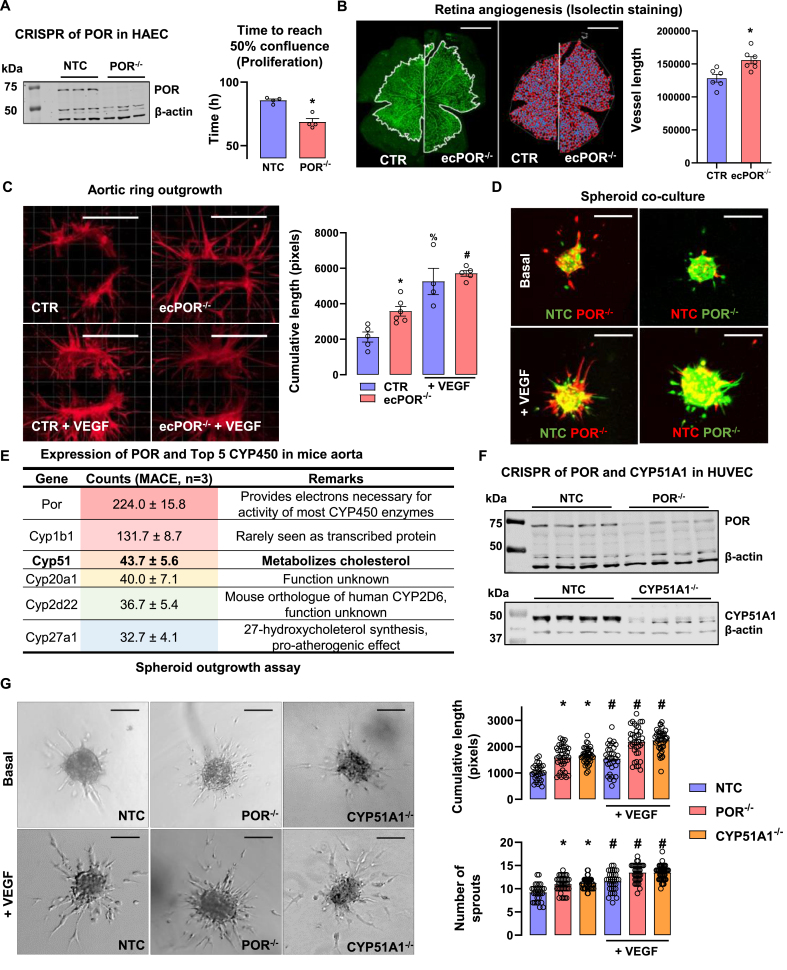
**Endothelial-specific knockout of POR induces angiogenesis**. A: Representative Western blot for POR in NTC and POR^−/−^ CRISPR/cas9 in HAEC. Proliferation assay (IncuCyte) of NTC and POR^−/−^ HAEC. Time to reach 50 % confluence plot. ∗p < 0.05 NTC versus POR^−/−^, Mann Whitney-Test. B: Retina angiogenesis (isolectin-stained) in neonatal mice as indicated (post natal day 7) from CTR and ecPOR^−/−^ mice with quantification; Scale bar: 1 mm ∗p < 0.05 NTC versus POR^−/−^, Mann Whitney Test C: *Ex vivo* endothelial cell outsprouting from aortic segments isolated from CTR and ecPOR^−/−^ mice with quantification under basal and VEGF (20 ng/mL) treatment; Scale bar: 1 mm ∗ p < 0.05 NTC versus POR^−/−^, %<0.05 NTC versus NTC + VEGF, #<0.05 POR^−/−^ versus POR^−/−^ VEGF, One-way ANOVA, Tukey correction. D: Confrontation spheroid assay with NTC and POR^−/−^ HUVEC (green or red as pseudo colour) with and without VEGF-A (30 ng/mL); Scalebar: 200 μM. E: CYP450 enzymes expressed in mouse aorta (MACE-seq, average ± STDEV.P, from n = 3). F: Western blotting for CYP51A1 in HUVEC CRISPR/cas9. G: Spheroid outgrowth assays using NTC, POR^−/−^, and CYP51A1^−/−^ HUVEC with and without VEGF-A (30 ng/mL) as indicated with quantification; Scalebar: 100 μM ∗p < 0.05 as compared to NTC without VEGF-A and #p < 0.05 as compared to NTC with VEGF-A, one-way ANOVA, Bonferroni correction.

To examine the role of POR in normal endothelial cell function, proliferation was evaluated using non-targeted control (NTC) and POR^−/−^ cells (CRISPR/Cas9-mediated knockout in HAEC). Deletion of POR significantly increased proliferation rate as compared to NTC cells so that the time to reach 50 % of confluency was 20 % shorter for POR^−/−^ cells (Fig. 1A).

Similar observations were made *in vivo*: Retina angiogenesis *in vivo* (neonatal retina model) using CTR and inducible, endothelial-specific knockout mice of POR (ecPOR^−/−^) showed that the later had a significantly increased vascularization as compared to CTR (Fig. 1B). This enhanced angiogenesis was also confirmed in an *ex vivo* aortic ring assay, where ecPOR^−/−^ aortae exhibited increased sprouting both under basal and VEGF-stimulated conditions as compared to CTR mice (Fig. 1C). These results identify POR as a negative regulator of angiogenesis. To further characterize this phenotype, confrontation spheroid assays were performed with color-coded NTC and POR^−/−^ HUVEC. When co-cultured, POR^−/−^ cells preferentially positioned at the leading part of the sprout as compared to the controls. These effects were observed under basal and VEGF-stimulated conditions (Fig. 1D).

Given that the only known function of POR is to provide electrons to CYPs, we reasoned that the pro-angiogenic effects of POR deletion are mediated through one of its redox CYP partners. Analysis of a previously published RNA-sequencing data set from mouse aortae [12] identified the cholesterol producing CYP51A1 as one of the most abundantly expressed CYP450 isoenzyme in EC (Fig. 1E). Thus, we hypothesized that the effects of POR deletion in angiogenesis might be a consequence of an inactive of POR/CYP51A1 system. To investigate this, CYP51A1 was deleted in HUVEC with CRISPR/Cas9 (Fig. 1F). Spheroid assays demonstrated that CYP51A1^−/−^ cells phenocopied the increased sprouting of POR^−/−^ cells (cumulative length and number of sprouts) (Fig. 1G, Suppl. Fig. 1A). To address whether migration drives the pro-angiogenic phenotype observed in POR^−/−^ and CYP51A1^−/−^ cells, a wound scratch assay was performed. There was, however, no difference in the wound closure between NTC and both knockout cell lines **(**Suppl. Fig. 2**)**, indicating that POR/CYP51A1 depletion does not enhance migratory capacity.

Altogether, these findings indicate that the angiogenic phenotype of POR deletion is at least in part mediated via disruption of CYP51A1-dependent cholesterol biosynthesis, supporting the notion that cholesterol availability tightly regulates angiogenic capacity.

### 3.2 Knockout of POR in endothelial cells disrupts cholesterol biosynthesis and triggers compensatory uptake mechanisms

**Fig. 2:**
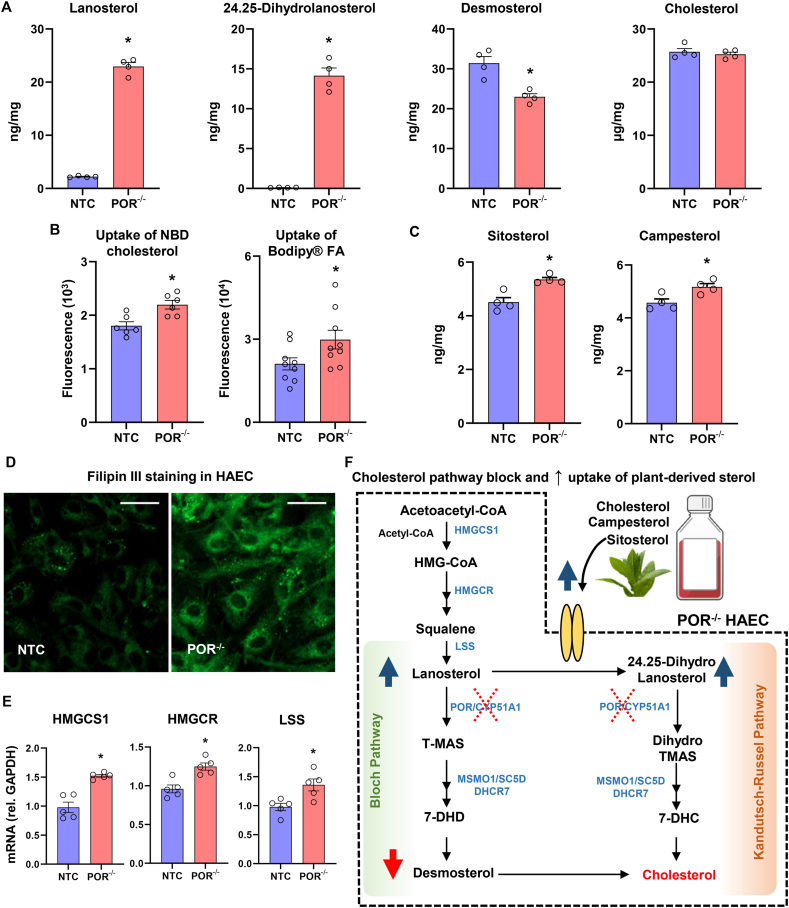
**Deletion of POR in endothelial cells disrupts cholesterol biosynthesis and triggers compensatory uptake mechanisms**. A: Measurements of cholesterol and its metabolites by GC/FID from HAEC NTC and POR^−/−^,∗p < 0.05 NTC versus POR^−/−^, Mann Whitney. B: Uptake of fluorescent NBD-cholesterol,∗p < 0.05 NTC versus POR^−/−^, Mann Whitney Test; uptake of Bodipy-FA in NTC and POR^−/−^ HAEC,∗p < 0.05 NTC versus POR^−/−^, T-test. C: Intracellular levels of plant-derived sterols (sitosterol, campesterol) as an indicative of uptake from the FCS contained in the growth media,∗p < 0.05 NTC versus POR^−/−^, Mann Whitney Test. D: Confocal images of Filipin III staining (free cholesterol, indicating its uptake by cells) in NTC and POR^−/−^ HAEC; Scale bar: 20 μM. E: RT-qPCR analysis in HAEC for genes of cholesterol synthesis,∗p < 0.05 NTC versus POR^−/−^, Mann Whitney Test. F: Schematic of cholesterol biosynthesis pathways highlighting POR/CYP51A1 involvement. HMGCS1: 3-hydroxy-3-methylglutaryl-CoA synthase 1; HMGCR: 3-hydroxy-3-methylglutaryl-CoA reductase; LSS: lanosterol synthase; T-MAS: testis meiosis-activating sterol; 7-DHD: 7-dehydrodesmosterol; 7-DHC: 7-dehydrocholesterol; MSMO1: methylsterol monooxygenase 1; SC5D: sterol-C5-desaturase; DHCR7: 7-dehydrocholesterol reductase.

To determine whether POR deletion affects endothelial lipid biosynthesis, cholesterol and its precursors were determined. GC-MS-SIM analysis of sterol intermediates revealed a strong accumulation of lanosterol and 24.25-dihydrolanosterol in POR^−/−^ cells compared to NTC, whereas desmosterol was significantly decreased (Fig. 2A). Similar effects were observed upon endothelial deletion of CYP51A1^−/−^ **(**Suppl. Fig. 3**)**. This confirms that POR and CYP51A1 activity are required in late steps of the cholesterol biosynthetic pathway.

Interestingly, despite differences in cholesterol biosynthesis intermediates, total cholesterol levels remained unchanged upon deletion of POR in EC. The most probable explanation for this is that cholesterol uptake compensates for the observed metabolic block so that more lipids of the cell culture medium were incorporated. Indeed, uptake of fluorescent NBD-cholesterol was significantly increased in POR^−/−^ cells as compared to controls (Fig. 2B). Likewise, the uptake of BODIPY-labeled fatty acids was also increased upon deletion of POR (Fig. 2B). Consistently, GC-FID analysis demonstrated elevated intracellular levels of the plant-derived sterols campesterol and sitosterol (Fig. 2C) in POR^−/−^ cells. These sterols are present in the foetal calf serum and can enter cells via Niemann-Pick C1-Like 1 (NPC1L1) or LDL-mediated uptake pathways [25]. Furthermore, unesterified (free) cholesterol which indicates cholesterol uptake by cells was also increased upon POR deletion as demonstrated by filipin III staining in HAEC NTC and POR^−/−^ (Fig. 2D). The stronger filipin signal in POR^−/−^ cells as compared to NTC likely reflects redistribution of cholesterol within cellular membranes due to augmented uptake. Altogether, these findings indicate that POR^−/−^ cells compensate for the reduced *de novo* synthesis by enhancing import of exogenous sterols.

### 3.3 Knockout of POR activates the Sterol Regulatory Element Binding Transcription Factor 2 (SREBP2) signalling, which is pro-angiogenic

**Fig. 3:**
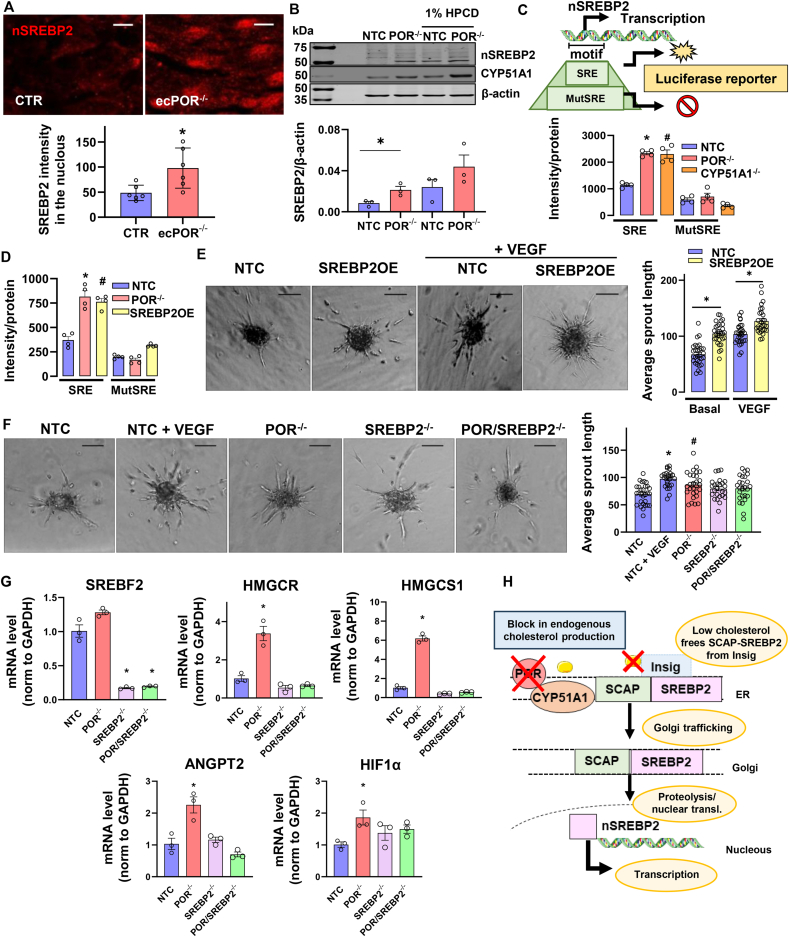
**Knockout of POR activates SREBP2 signaling which promotes angiogenesis.** A: E*n Face* of aorta from CTR and ecPOR^−/−^ mice using a nuclear specific SREBP2 antibody and quantification; Scale bar: 10 μM, ∗p < 0.05 NTC versus POR^−/−^, Mann Whitney Test. B: Western blotting showing nSREBP2 activation in CRISPR/cas9 cells treated or not with 1 % HPCD, ∗p < 0.05 NTC versus POR^−/−^, *t*-test unpaired. C: Luciferase reporter assay for SREBP2 using a construct containing a SRE and a muted construct as negative control as depicted in the figure and measurement in NTC, POR^−/−^ and CYP51 ^−/−^ HUVEC ∗ p < 0.05 NTC versus POR^−/−^, #p < 0.05 NTC versus CYP51A1^−/−^, Mann Whitney Test. D: Luciferase reporter assay for SREBP2 using a construct containing a SRE (Sterol Regulatory Element) and a muted construct (MutSRE) as negative control as depicted in the figure and measurement in NTC, POR^−/−^ and SREBP2 over-expression (SREBP2OE) HUVEC ∗ p < 0.05 NTC versus POR^−/−^, #p < 0.05 NTC versus SREBP2OE, Mann Whitney Test. E-F: Spheroid outgrowth assay in NTC, SREBP2OE; Scalebar: 100 μM, ∗p < 0.05 NTC versus SREBP2OE (E) and POR^−/−^, SREBP2^−/−^ and POR/SREBP2^−/−^; Scalebar: 100 μM, ∗p < 0.05 NTC versus NTC VEGF and #p < 0.05 NTC versus POR^−/−^ (G). F: RT-qPCR for genes as indicated in HAEC with low FCS (0.5 %, 4 h) showing that the nSREBP2 transcriptional activation is dependent on POR. ∗p < 0.05 NTC versus knockouts as indicated in each panel, Mann Whitney Test, unpaired. H: Schematics of how deletion of POR leads to SRBEP2 activation.

Given that deletion of POR inhibits endogenous cholesterol synthesis without changing total cholesterol levels, we hypothesized that intracellular cholesterol sensing is altered in POR^−/−^ cells and that the increased angiogenesis is a consequence of the activation of this sensing system. Indeed, we found strong evidence for an activation of a compensatory system: Transcriptional analysis by RT-qPCR revealed that the block in the POR/CYP51A1 step of cholesterol synthesis in POR^−/−^ cells was compensated by an upregulation in the expression of their upstream enzymes HMGCS1 (3-Hydroxy-3-Methylglutaryl-CoA Synthase 1), HMGCR (3-Hydroxy-3-Methylglutaryl-CoA Reductase), and LSS (Lanosterol Synthase) (Fig. 2E and F). Interestingly, recent work showed that an increase in LSS expression is associated with an activation of angiogenic pathways in cancer cells [26]. *Ex vivo* aortic *en face* immunostaining from mice showed a pronounced nuclear localization of SREBP2 (nSREBP2) in ecPOR^−/−^ mice as compared to CTR (Fig. 3A). In HAEC, Western blot analysis revealed an increased nSREBP2 upon treatment of cells with 1 % HPCD (2-Hydroxypropyl-β-cyclodextrin, which extracts cholesterol from cell membranes). This effect was also observed without HPCD in POR^−/−^ cells and was more pronounced upon HPCD treatment (Fig. 3B). These findings are consistent with SREBP2 activation as a compensatory mechanism to counteract changes in endogenous sterol production as well as a decrease in membrane cholesterol. To provide direct evidence for SREBP2 activation, we developed a luciferase reporter assay using a sterol-responsive element (SRE). This confirmed that transcriptional activity of SREBP2 was significantly increased in POR^−/−^ cells and in CYP51A1^−/−^ cells and mutation of the SRE binding element prevented this effect (Fig. 3C).

To evaluate whether nSREBP2 alone promotes angiogenesis, we overexpressed the protein using a lentiviral system in HUVEC. Increased activity of nSREBP2 upon transduction was confirmed by luciferase reporter assay. Interestingly, overexpression of nSREBP2 increased activity to a similar extent as deletion of POR^−/−^ in EC (Fig. 3D). Importantly, also concerning angiogenesis, overexpression of nSREBP2 phenocopied the effect of POR deletion and increased angiogenesis (Fig. 3E, Suppl. Fig. 1B). Importantly, genetic deletion of SREBP2 abrogated the pro-angiogenic effect of POR deletion (Fig. 3F, Suppl. Fig. 1C). These effects extended to the downstream target genes of nSREBP2 as determined by RT-qPCR (SREBF2, HMGCR, HMGCS1, ANGPT2 and HIF1α). Deletion of POR upregulated expression of these genes and deletion of SREBP2 also abrogated this effect (Fig. 3G). These results indicate that SREBP2 activation (Fig. 3H) is both necessary and sufficient to mediate the pro-angiogenic effects of POR loss.

### 3.4 Deletion of POR upregulates endothelial genes related to angiogenesis

**Fig. 4:**
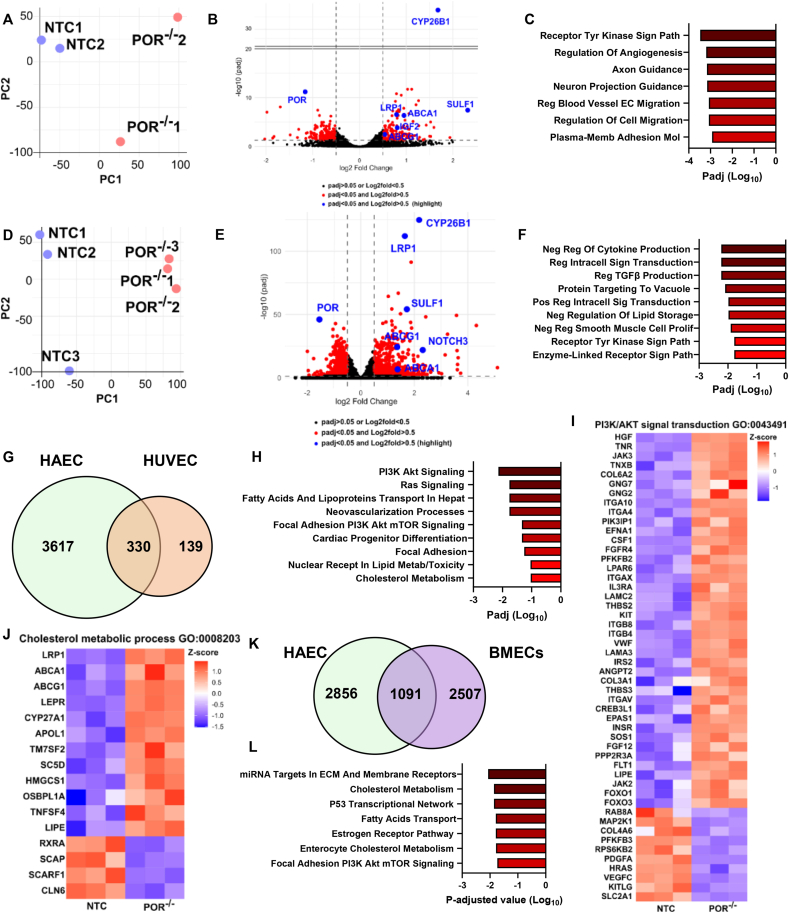
**Endothelial deletion of POR in HAEC upregulates genes related to angiogenesis**. A: PCA plot of RNAseq from NTC and POR^−/−^ HUVEC reporter cell lines co-cultivated after being FACS sorted to separate cyan and green populations, n = 2. B: Volcano plot of significant differentially expressed genes in POR^−/−^ versus NTC). Cholesterol-related genes are highlighted in blue. C: Pathway enrichment analysis for top upregulated GO Biological functions. D: PCA plot of RNAseq from NTC and POR^−/−^ HAEC; n = 3. E: Volcano plot of significant differentially expressed genes (POR^−/−^ versus NTC). Cholesterol-related genes are highlighted in blue. F: Pathway enrichment analysis for top upregulated GO Biological functions. G: Overlap of genes from RNAseq of HUVEC and HAEC. H: Pathway enrichment analysis for top upregulated GO Biological functions (POR^−/−^ versus NTC) from the overlapping genes in both HUVEC and HAEC. I-J: Heatmap of genes that annotate for PI3K/AKT signal transduction and cholesterol metabolic pathway from the HAEC RNAseq. K: Overlap of differentially expressed genes (padj <0.05) between POR^−/−^ HAEC and POR-deficient BMECs from GSE202485. L: Gene set enrichment analysis of the shared genes in K using Enrichr (WikiPathways 2024, Human) revealed cholesterol and PI3K signaling as the top enriched pathway.

To investigate how alterations in gene expression are associated with the increased angiogenesis in response to POR deletion transcriptomic was performed. As POR deletion impacts on proliferation and cell density impacts gene expression, a mixed culture of reporter control and POR-deficient cells was used to allow for cell sorting. RNAseq was performed from the reporter cell lines which were co-cultured (Fig. 4A–C) as well as from HAEC (Fig. 4D and E) NTC and POR^−/−^. In the different cell batches analysed, the NTC and POR^−/−^ genotypes segregated in principal component analysis (Fig. 4A–D). GO term annotation of upregulated genes show a significant signature for regulation of angiogenesis and receptor tyrosine kinase signalling (Fig. 4C–F). Importantly, there was an overlap of over 70 % of the significantly expressed genes between HAEC and HUVEC (Fig. 4G and H).

Although several genes annotated to cholesterol metabolism were upregulated in POR^−/−^ cells as compared to NTC (Fig. 4J), they did not rank as the most significantly altered genes. Rather, PI3K/AKT-related genes, which are associated with angiogenesis, cell growth, survival, and proliferation. To assess the consistency of POR-dependent transcriptional regulation across other endothelial cell types, we reanalysed RNAseq data from a published POR-deficient brain microvascular endothelial cells (BMEC) (GSE202485, [23]) and compared it to our HAEC POR^−/−^ dataset. Differential expression analysis revealed that 1,091 genes were commonly regulated in both datasets (Fig. 4K). Gene set analysis of the shared DEGs revealed that both cholesterol and PI3K-related signalling pathways were significantly enriched, suggesting that PI3K/AKT axis activation is a conserved downstream effect of POR deficiency in EC (Fig. 4L).

### 3.5 Knockout of POR promotes the activation of pro-angiogenic kinases

**Fig. 5:**
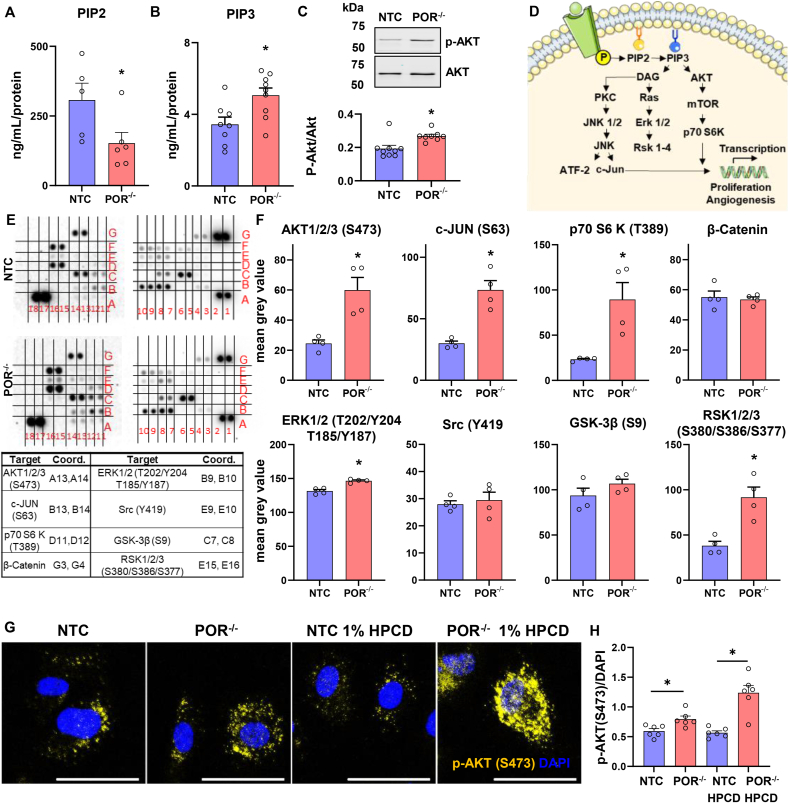
**POR deletion activates PI3K signalling**. Quantification of PIP2 (A) and PIP3 (3) levels by ELISA,∗p < 0.05 NTC versus POR^−/−^, Mann Whitney. C: Western blotting and quantification for AKT-phosphorylation (S473) (P-AKT) under basal conditions. ∗p < 0.05 NTC versus POR^−/−^, Mann Whitney. D: Schematics of Tyrosine-Kinase activation. E-F: Phospho-kinase array kit assay with coordinates of targets and quantification. ∗p < 0.05 NTC versus POR^−/−^, Mann Whitney. G: Representative images from immunofluorescence showing P-AKT (S473) (yellow) and DAPI (blue) in NTC and POR^−/−^ and 1 %HPCD treated cells (1h); Scale bar: 40 μM, with respective quantification (F),∗p < 0.05 NTC versus POR^−/−^, Mann Whitney Test.

Thus, we studied a potential functional activation of the PI3K/AKT angiogenic pathways. Since tyrosine-kinase receptor activation triggers a conversion of PIP2 to PIP3, these two phosphoinositides were measured by ELISA in NTC and POR^−/−^ cells. These measurements demonstrated a significant increase in PIP3 levels and a significant reduction in PIP2 in POR^−/−^ cells relative to NTC, consistent with enhanced PI3K activity (Fig. 5A and B). Western blot analysis confirmed increased phosphorylation of AKT (Ser473) in POR^−/−^ as compared to NTC under basal conditions (Fig. 5C). To comprehensively profile the downstream signalling changes, a phospho-kinase array was performed using HAEC lysates (Fig. 5D–F, Suppl. Fig. 4). This revealed a significantly increased phosphorylation of AKT (S473), p70 S6 kinase (T389), c-Jun (S63), and RSK1/2/3 (S380/S386/S377), key nodes of the PI3K/AKT/mTOR and MAPK pathways.

Immunofluorescence analysis confirmed increased basal pAKT levels in POR^−/−^ cells as compared with NTC (Fig. 5G and H). Furthermore, treatment with 1 % HPCD, which is known to activate SREBP2 signalling [27], further enhanced pAKT levels, indicating that acute perturbation of sterol homeostasis amplifies PI3K/mTOR pathway activity. Notably, AKT phosphorylation (S473), seen in WB, was elevated even after additional knockout of SREBP2 in POR^−/−^/SREBP2^−/−^ cells, suggesting that basal PI3K/AKT activation is not dependent on SREBP2 **(**Suppl. Fig. 5**)**. However, despite persistent AKT activation, the enhanced sprouting phenotype observed in POR^−/−^ cells was lost upon SREBP2 deletion, indicating that AKT activation alone is not sufficient to promote angiogenesis in the absence of SREBP2-dependent transcriptional reprogramming. Collectively, these findings suggest that disruption of intracellular sterol homeostasis activates PI3K/AKT/mTOR signalling and SREBP2-dependent gene expression, both of which contribute to the pro-angiogenic endothelial phenotype following POR deletion.

## 4 Discussion

In this study we demonstrate that endogenous cholesterol synthesis is essential for normal endothelial cell function and how its disruption influences endothelial cell behaviour, in particular to angiogenesis. This was, so far, a poorly explored aspect in vascular biology likely for the following reasons: i. The focus on cholesterol metabolism has been mainly on its deleterious effects such as in atherosclerosis; ii. The fact that about 50 % of total cholesterol synthesis in humans occurs in the liver [13] somehow undermines the importance of endogenous cholesterol synthesis in vascular cells; iii. EC can tightly regulate cholesterol and fatty acid uptake from the blood stream [[28], [29], [30]]. In fact, the effects of statins, as inhibitors of HMGCR, on angiogenesis have been intensely studied and show a dual effect: Concentrations in the therapeutic range enhance endothelial cell proliferation, migration, differentiation, and survival. In contrast, very high concentrations of statins suppress angiogenesis by inducing endothelial apoptosis [31]. Whereas the protective effects are mediated by reduced geranyl-geranylation, the deleterious effects are a consequence of mitochondrial dysfunction due to insufficient lipid-anchoring of ubiquinone in the matrix membrane [32]. The reaction catalysed by POR/CYP51A1 in cholesterol biosynthesis is downstream of that catalysed by HMGCR (which is rate limiting) [33] and has not been pharmacologically exploited. Thus, it was unexpected that POR deletion in EC enhanced angiogenesis. In fact, we had rather expected the opposite effect as depletion of POR lowers EET levels [12] and EETs are thought to be proangiogenic [9]. Therefore, a role for EETs generated by endothelial CYP2C8 and CYP2C9 does not generate the dominant phenotype observed in the present study. Interestingly, CYP26B1 was among the upregulated CYP enzymes in POR-deficient cells. As CYP26B1 is a major retinoic acid-metabolizing enzyme [34] its induction may alter retinoic acid availability, which has been reported to suppress endothelial proliferation and angiogenesis [35,36]. While these observations suggest that additional CYP-dependent pathways may reflect a compensation for POR deletion, the close phenotypic overlap between POR and CYP51A1 deletion strongly supported cholesterol biosynthesis as the primary driver for the observed pro-angiogenic phenotype.

Increased angiogenesis upon deletion of POR was demonstrated *in vivo* in the neonatal retina model and *ex vivo* in the aortic ring outgrowth assay. Notably, these effects occurred independently of any supplemented VEGF. To validate these results, we used the spheroid outgrowth assay with primary EC and demonstrated that knockout of both POR and CYP51A1 under basal conditions is pro-angiogenic.

These findings prompted us to explore how cholesterol metabolism might underlie the observed pro-angiogenic phenotype. Changes in cholesterol biosynthesis were assessed by GC-MS-SIM. A blockage in the pathway by deletion of POR or CYP51A1 led to an accumulation of the cholesterol precursor lanosterol and 24.25-dihydrolanosterol confirming the shutdown of the neo-cholesterol synthesis in EC. This blockage was further demonstrated by the converse reduction of the downstream metabolite desmosterol. A valid consideration is whether the pro-angiogenic phenotype in the present model is driven by depletion of desmosterol or an accumulation of lanosterol. The latter has been reported to influence cellular signalling independently of cholesterol levels [37] suggesting that disruption of sterol flux, rather than simple cholesterol depletion alone, may alter signalling. In fact, lanosterol can insert into lipid bilayers, but it has different biophysical features as compared to cholesterol. Lanosterol is less miscible and less well packed in membranes than cholesterol, so it perturbs membrane organization and receptor signalling differently [38,39].[PMID: 16905603] (PMID: 10653627).

Remarkably, total cholesterol levels themselves remained unchanged as EC compensated this imbalance by increasing cholesterol/lipid uptake, as supported by the uptake assays. The increase in uptake, however, did not appear to fully substitute for endogenously synthesized cholesterol, prompting cells to engage mechanisms aimed at restoring sterol homeostasis, most notably via nSREBP2 activation, which was demonstrated by Western blot analysis, *en face* immunostaining and a luciferase reporter assay.

Strikingly, overexpression of nSREBP2 alone led to a significant increase in angiogenic sprouting (spheroid assays). This directly links SREBP2 activity to the promotion of angiogenesis. In previous studies on SREBP2 in EC it has been shown that it mediates inflammatory stress and response to cytokines [40,41]. Regarding angiogenesis, the association to SREBP2 was indirect, as treatment of EC with VEGF activated SREBP2 activity, increasing expression of HMGCR and thus promoting angiogenesis [42,43]. Importantly, the POR^−/−^ induced angiogenesis was abrogated by knocking out SREBP2^−/−^ as shown in the POR^−/−^ and SREBP2^−/−^ double-knockout cells. These results underscore SREBP2 as a central mediator of the angiogenic phenotype observed in POR-deficient cells.

SREBP2 activation has been shown to be associated with reprogramming of lipid metabolism and the association between SREBP2 and AKT signalling has been previously demonstrated but in the context of oncogenic signalling [[44], [45], [46]]. RNAseq analysis of POR^−/−^ in HAEC and HUVEC revealed significant alterations in pathways related to sterol regulation, membrane-associated tyrosine kinase signalling, and angiogenesis, compared to NTC cells. The downstream signalling was further activated and confirmed through the ELISA-based measurements with an increase in PIP3 and a corresponding decrease in PIP2 in the POR^−/−^ HAEC, indicating enhanced PI3K activity and further supporting AKT pathway activation, which was also confirmed through Western blotting and immunofluorescence. Furthermore, phosphorylation levels of other tyrosine kinases such as p70 S6 kinase, c-Jun, and RSK1/2/3 were also increased in POR^−/−^ HAEC, as shown by a phospho-kinase array. It has been reported that enhanced cholesterol uptake can indirectly activate PI3K signalling [47,48] through lipid-mediated feedback on SREBP2, though direct mechanisms are not clearly established yet. AKT phosphorylation has also been associated to SREBP2 activation through the mTORC1 pathway. Thus, the observed pro-angiogenic effects of POR and CYP51A1 deficiency support the idea that disruption of cholesterol biosynthesis activates broader signalling networks that enhance endothelial angiogenic responses. Given the central role of membrane sterols in organizing signalling platforms and regulating receptor activity, the consequences of altered sterol homeostasis are unlikely to be restricted to angiogenesis as membrane sterol composition is known to regulate also endothelial barrier integrity and nitric oxide-dependent vascular function [49,50].

**Fig. 6:**
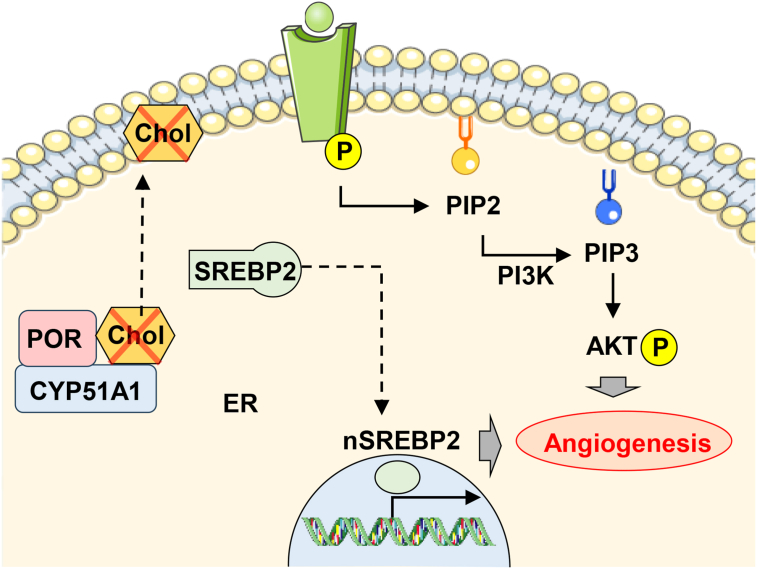
**Endogenous cholesterol synthesis limits angiogenesis**. Endothelial deletion of POR along with CYP51A1 inhibits endogenous cholesterol synthesis and promotes angiogenesis a process associated with activation of SREBP2 and PIP3-dependent kinases.

What are translational implications of the present work? Several compounds, including the antifungal drug itraconazole, a known CYP51A1 inhibitor, have been proposed for repurposing as anti-angiogenic agents in cancer therapy. At first glance, this appears paradoxical, given that genetic disruption of the POR/CYP51A1 axis enhanced angiogenesis in our study. However, chronic deletion of POR/CYP51A1 induces compensatory metabolic adaptation, including SREBP2 activation, increased sterol uptake, and increased kinase signaling (Fig. 6), whereas pharmacological inhibition with itraconazole is typically acute. Moreover, itraconazole's anti-angiogenic effects are thought to depend in part on pleiotropic actions beyond CYP51A1 inhibition, including suppression of VEGFR2 trafficking and glycosylation [51]. Our findings, therefore, suggest a potential metabolic adaptation in response to cholesterol biosynthesis perturbation instead of sole changes in cholesterol levels. The present results identify endothelial sterol sensing as a regulator of angiogenic signaling and raise the possibility that selective modulation of the POR/CYP51A1-SREBP2 axis could provide opportunities to therapeutically regulate angiogenesis in cardiovascular disease, cancer, and neovascular disorders.

Whereas CYP51A1 inhibitors have clinical use, SREBP2 inhibitors, such as Fatostatin (a diarylthiazole that binds SCAP, blocking ER-to-Golgi translocation of SREBP to the nucleus) has only been used as a research compound, not as a clinical drug yet.

Regarding human genetic conditions that modulate POR, CYP51A1 and SREBP2, the main associated diseases can not be exclusively linked to the proposed mechanism observed in this study. POR is highly associated with Antley Bixter syndrome, a rare congenital craniosynostosis/dysmorphia syndrome characterized by distinctive craniofacial and skeletal malformations. However, there are SNPs within the POR gene associated with cardiovascular diseases (rs181538359 and rs113454523) [52]. CYP51A1 is highly associated with cataract and SREBP2 with neurodegenerative disease (https://platform.opentargets.org).

In conclusion, the disruption of cholesterol biosynthesis through POR or CYP51A1 deficiency activates a multifaceted response involving SREBP2 and kinase signalling pathways, which collectively drive enhanced angiogenesis (Fig. 6). These findings deepen our understanding of the interplay between metabolic regulation of cholesterol and endothelial function and open new avenues for understanding and targeting angiogenesis.

## Disclosures

The authors declare no conflict of interest that could potentially influence or bias the work.

## Source of funding

The study was funded by the Hessian research program LOEWE/2/18/519/03/11.001(0005)/124 Lipid Space. The 10.13039/501100001659Deutsche Forschungsgemeinschaft (RE 4360/2-1 to FR; 10.13039/501100021703Excellent Cluster Cardio-Pulmonary Institute EXS2026 to RPB). The 10.13039/501100005687Medicine Faculty of the Goethe University (Frankfurt, Germany). The German Center for Cardiovascular Research (DZHK), Partner Site Rhein-Main.

## CRediT authorship contribution statement

**Pedro F. Malacarne:** Data curation, Formal analysis, Investigation, Methodology, Writing – original draft. **Melina Lopez:** Data curation, Formal analysis, Investigation, Methodology. **Souradeep Chatterjee:** Data curation, Formal analysis, Methodology. **Niklas Herrle:** Data curation, Investigation, Methodology. **Lisa Weiss:** Methodology. **Stefan Günther:** Data curation, Formal analysis, Investigation, Methodology. **Timothy Osborne:** Methodology, Resources, Writing – review & editing. **Clemens Glaubitz:** Funding acquisition, Methodology. **Tobias Schmid:** Funding acquisition, Methodology, Writing – review & editing. **Nina Kurrle:** Methodology, Resources, Writing – review & editing. **Frank Schnütgen:** Methodology, Resources, Writing – review & editing. **Dieter Lütjohann:** Data curation, Investigation, Methodology, Writing – review & editing. **Ralf P. Brandes:** Conceptualization, Resources, Writing – original draft, Writing – review & editing. **Flávia Rezende:** Conceptualization, Funding acquisition, Investigation, Project administration, Resources, Supervision, Visualization, Writing – original draft, Writing – review & editing.

## Declaration of competing interest

The authors declare that they have no known competing financial interests or personal relationships that could have appeared to influence the work reported in this paper.

## Data Availability

No data was used for the research described in the article.

## Appendix A Supplementary data

To review GEO accession GSE326111:

Go to https://www.ncbi.nlm.nih.gov/geo/query/acc.cgi?acc=GSE326111.

Enter token qbcdewuyjtwfncz.
