# Supplementary material for: Endothelial cytochrome P450 -derived cholesterol limits angiogenesis
Source: Redox Biol. 2026 Jul 7;95:104289. doi: 10.1016/j.redox.2026.104289 (PMC13376976; doi:10.1016/j.redox.2026.104289)
Supplement: Multimedia component 2 [file mmc2.pptx]

## Slide 1
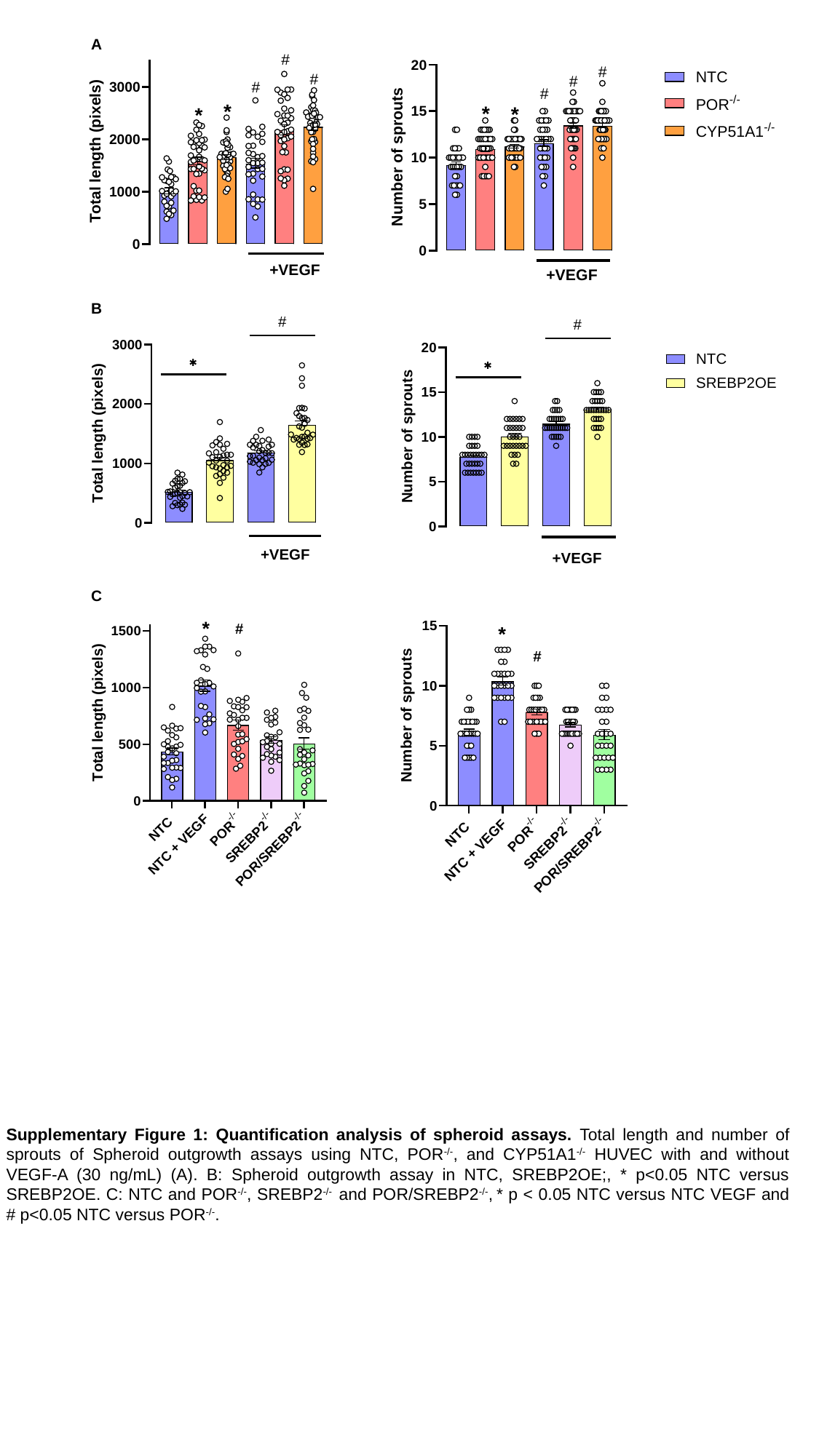

A
B
C
Supplementary Figure 1: Quantification analysis of spheroid assays. Total length and number of sprouts of Spheroid outgrowth assays using NTC, POR-/-, and CYP51A1-/- HUVEC with and without VEGF-A (30 ng/mL) (A). B: Spheroid outgrowth assay in NTC, SREBP2OE;, * p<0.05 NTC versus SREBP2OE. C: NTC and POR-/-, SREBP2-/- and POR/SREBP2-/-, * p < 0.05 NTC versus NTC VEGF and # p<0.05 NTC versus POR-/-.

## Slide 2
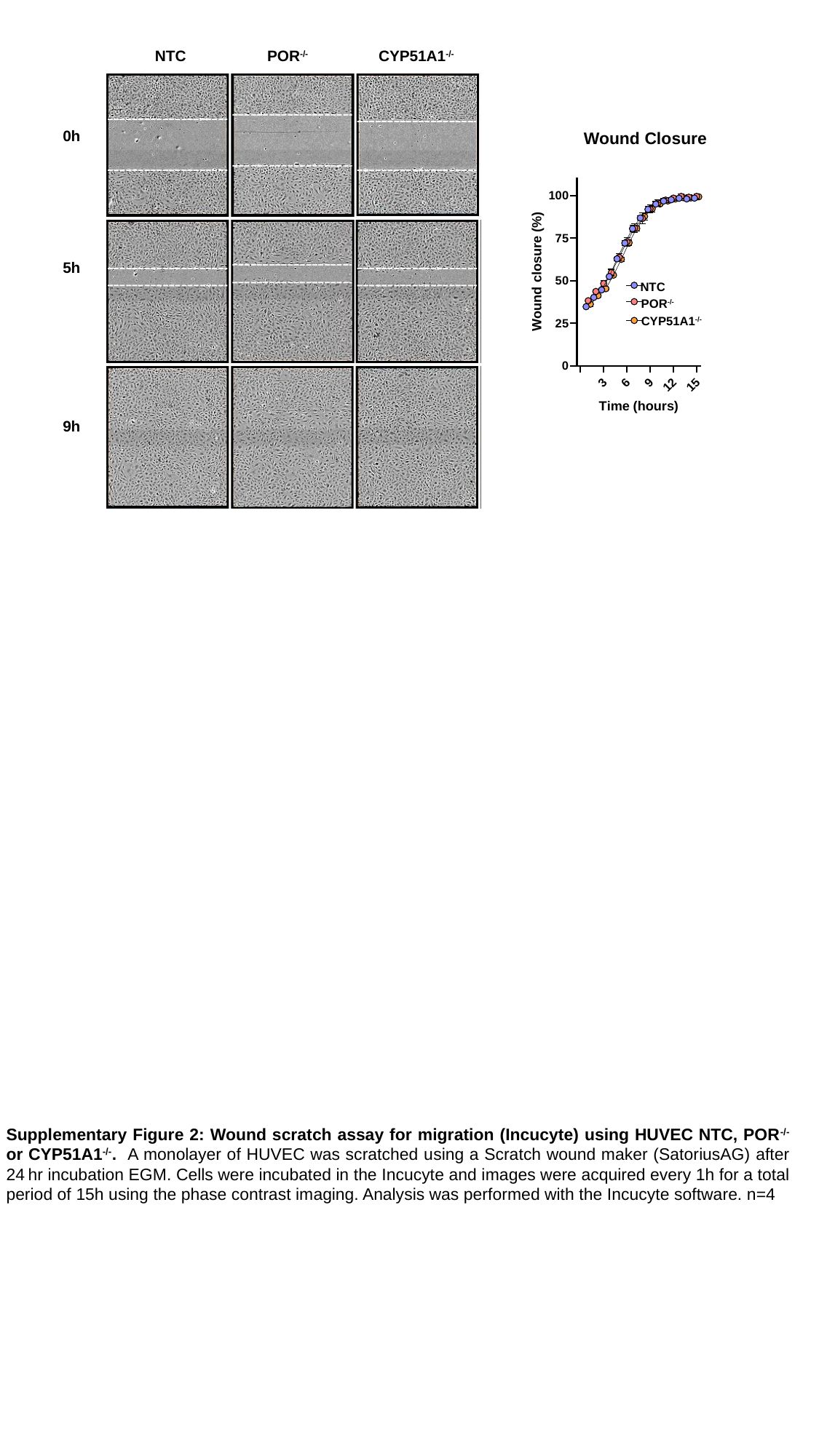

POR-/-
CYP51A1-/-
NTC
0h
Wound Closure
5h
NTC
POR-/-
CYP51A1-/-
9h
Supplementary Figure 2: Wound scratch assay for migration (Incucyte) using HUVEC NTC, POR-/- or CYP51A1-/-. A monolayer of HUVEC was scratched using a Scratch wound maker (SatoriusAG) after 24 hr incubation EGM. Cells were incubated in the Incucyte and images were acquired every 1h for a total period of 15h using the phase contrast imaging. Analysis was performed with the Incucyte software. n=4

## Slide 3
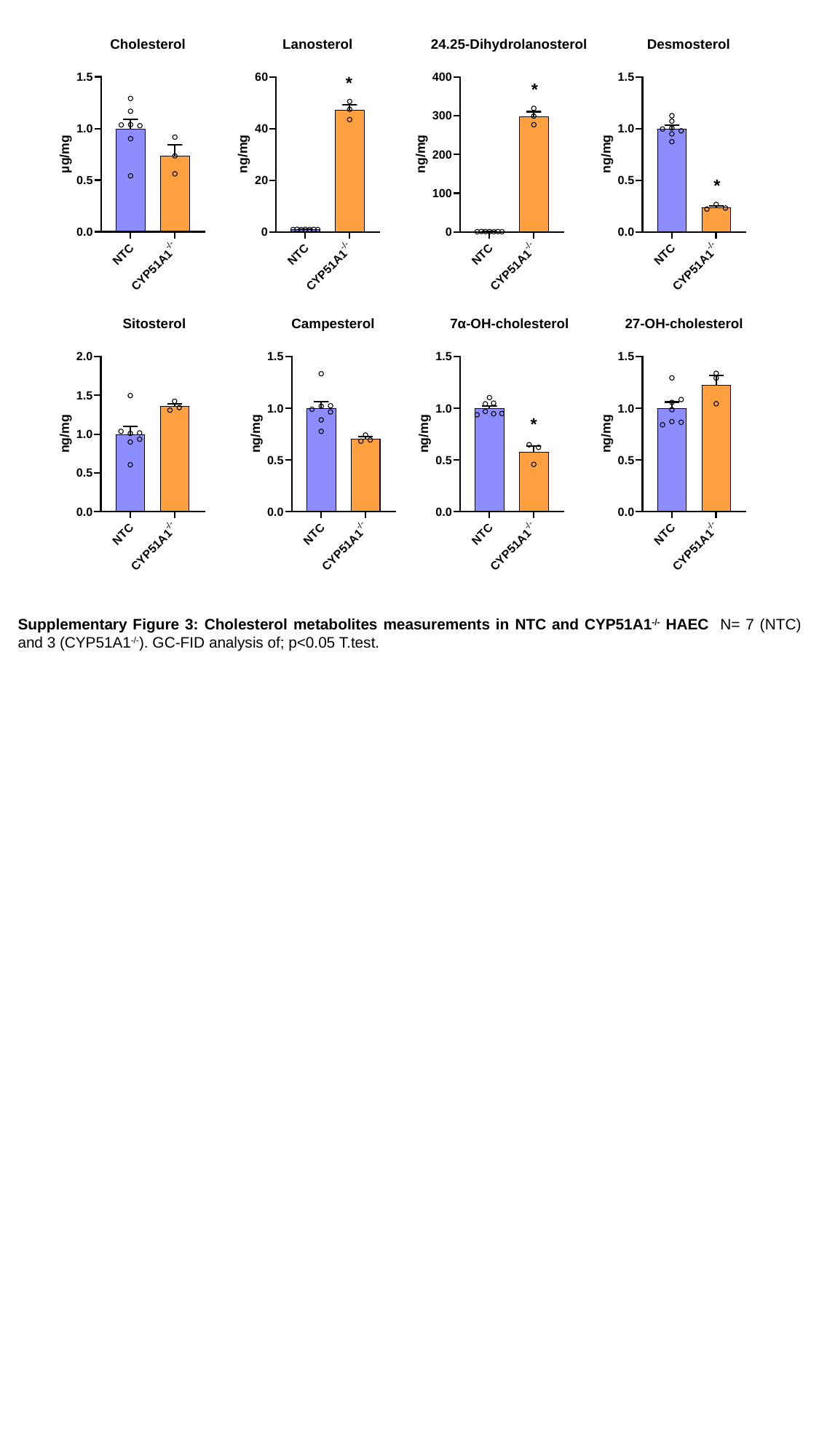

Lanosterol
Cholesterol
24.25-Dihydrolanosterol
Desmosterol
Sitosterol
Campesterol
7α-OH-cholesterol
27-OH-cholesterol
Supplementary Figure 3: Cholesterol metabolites measurements in NTC and CYP51A1-/- HAEC N= 7 (NTC) and 3 (CYP51A1-/-). GC-FID analysis of; p<0.05 T.test.

## Slide 4
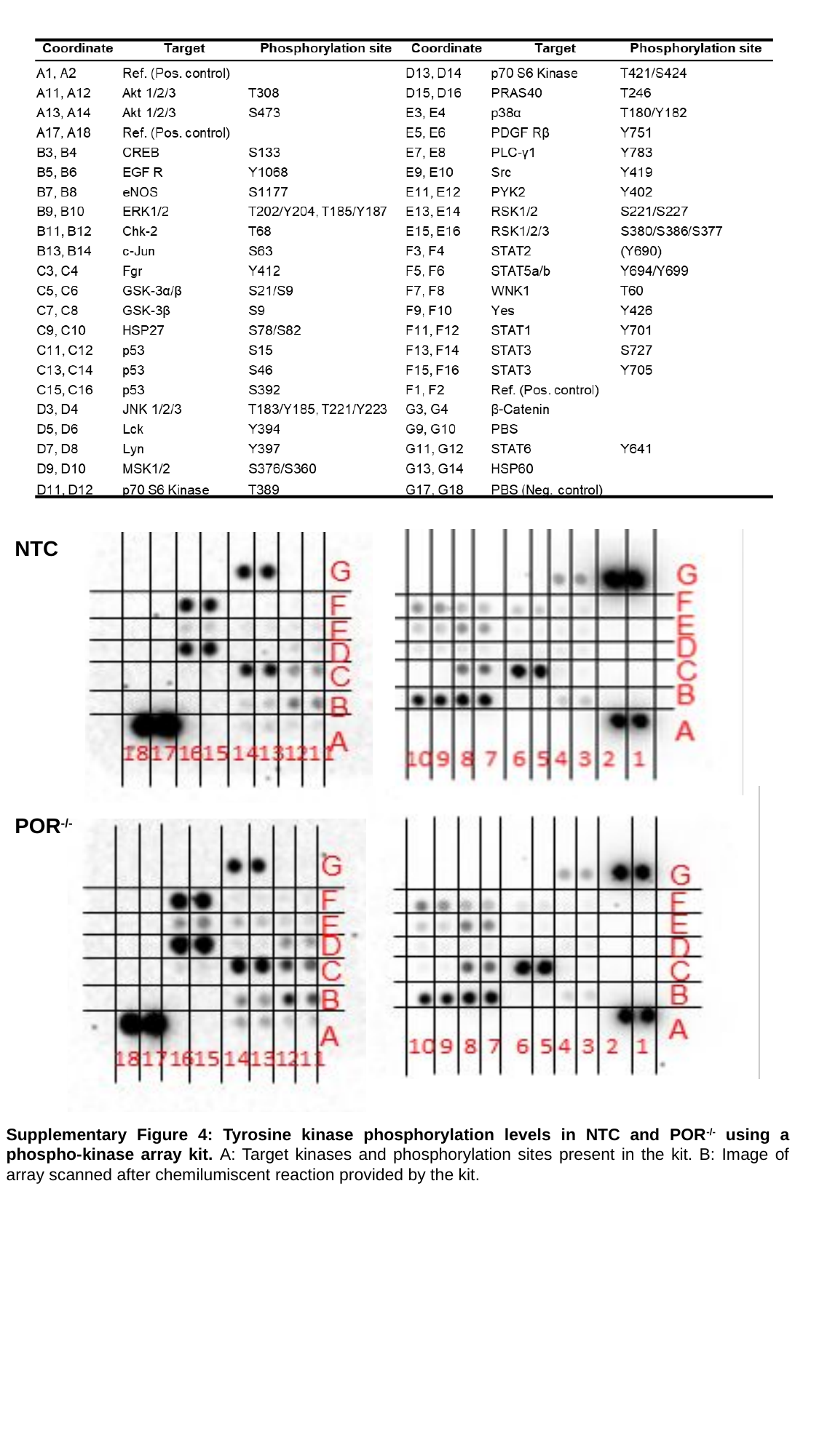

NTC
POR-/-
Supplementary Figure 4: Tyrosine kinase phosphorylation levels in NTC and POR-/- using a phospho-kinase array kit. A: Target kinases and phosphorylation sites present in the kit. B: Image of array scanned after chemilumiscent reaction provided by the kit.

## Slide 5
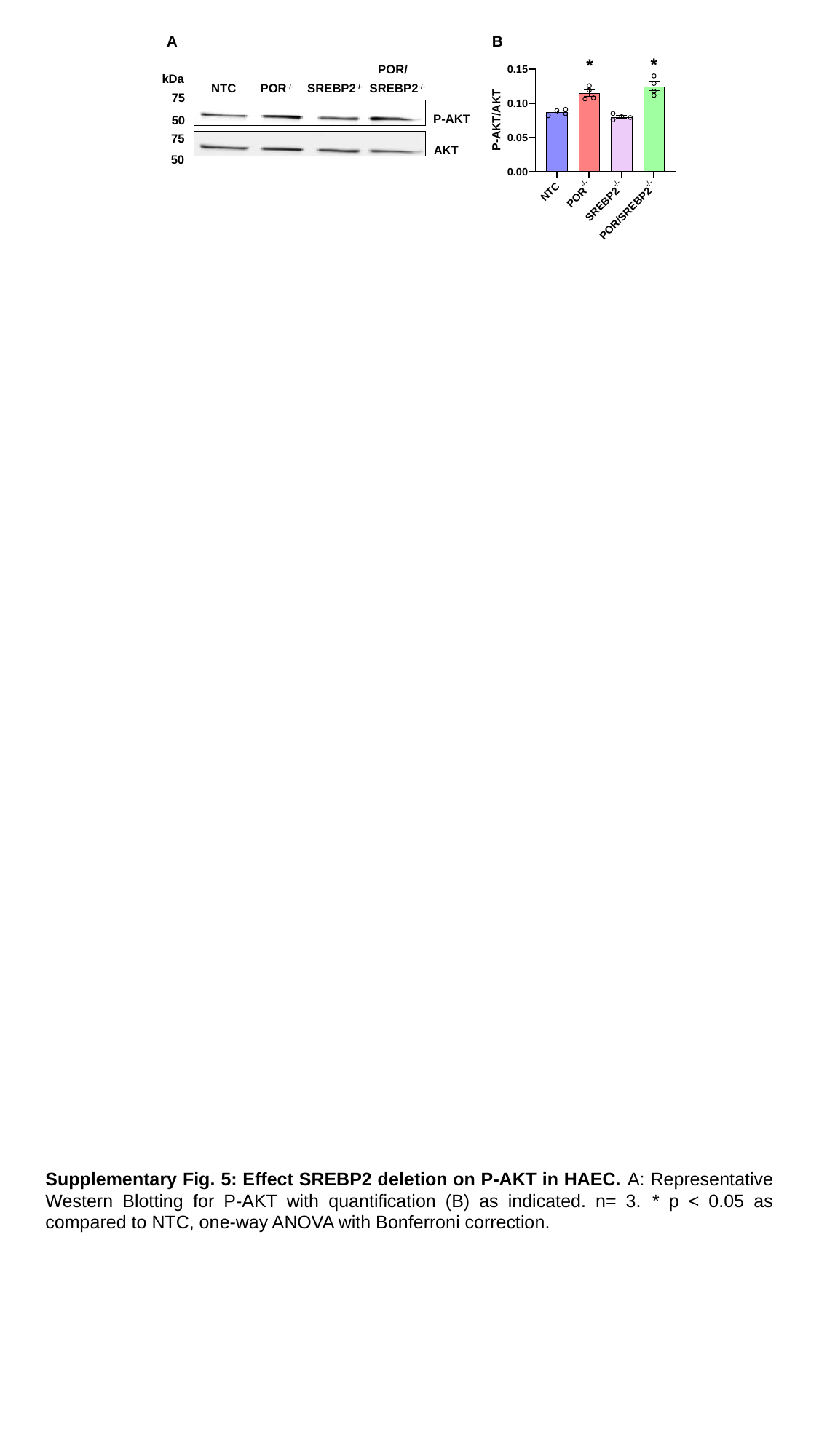

A
B
POR/
 NTC POR-/- SREBP2-/- SREBP2-/-
P-AKT
AKT
kDa
75
50
75
50
Supplementary Fig. 5: Effect SREBP2 deletion on P-AKT in HAEC. A: Representative Western Blotting for P-AKT with quantification (B) as indicated. n= 3. * p < 0.05 as compared to NTC, one-way ANOVA with Bonferroni correction.
